# Test Equating of the Medical Licensing Examination in 2003 and 2004 Based on the Item Response Theory

**DOI:** 10.3352/jeehp.2006.3.2

**Published:** 2006-07-08

**Authors:** Mi Kyoung Yim, Sun Huh

**Affiliations:** National Health Personnel Licensing Examination Board, Seoul, Korea.; 1 Department of Parasitology, College of Medicine and Institute of Medical Education, Hallym University, Chuncheon, Korea.

**Keywords:** Test Equating, Education, Medical, licensing Examination, Item Response Theory

## Abstract

The passing rate of the Medical Licensing Examination has been variable, which probably originated from the difference in the difficulty of items and/or difference in the ability level of examinees. We tried to explain the origin of the difference using the test equating method based on the item response theory. The number of items and examinees were 500, 3,647 in 2003 and 550, 3,879 in 2004. Common item nonequivalent group design was used for 30 common items. Item and ability parameters were calculated by three parametric logistic models using ICL. Scale transformation and true score equating were executed using ST and PIE. The mean of difficulty index of the year 2003 was -0.957 (SD 2.628) and that of 2004 after equating was -1.456 (SD 3.399). The mean of discrimination index of year 2003 was 0.487 (SD 0.242) and that of 2004 was 0.363 (SD 0.193). The mean of ability parameter of year 2003 was 0.00617 (SD 0.96605) and that of year 2004 was 0.94636 (SD 1.32960). The difference of the equated true score at the same ability level was high at the range of score of 200-350. The reason for the difference in passing rates over two consecutive years was due to the fact that the Examination in 2004 was easier and the abilities of the examinees in 2004 were higher. In addition, the passing rates of examinees with score of 270-294 in 2003, and those with 322-343 in 2004, were affected by the examination year.

## INTRODUCTION

The Medical Licensing Examination is the criterion-referenced test that the medical license is provided when examinees pass a certain level of knowledge or skills. Therefore, the annual examination must maintain equal level of item difficulty. If not, there might be a fluctuation in the passing rate. Although the passing rate was affected by item difficulty, the ability of the examinees in a year also may be another factor of influence to the passing rate. However, since the exam items are different to avoid the problem of item exposure and the population of examinees changed year by year, it is difficult to compare the scores directly. In one OECD country, the passing rate of Medical Licensing Examination was 90%. Recently there was a fluctuation in the passing rate, *i.e.* 86.6% in 2003 and 96.9% in 2004. This phenomenon may become an issue of debate by the interested groups such as medical students and medical teachers.

When there is such a discrepancy between passing rates over two years, it is necessary to know if it is originated by the difference in the item difficulty or by the difference in the abilities of examinees. To answer, we tried to apply test equating. Equating is the statistical process used to adjust scores on test forms so that scores can be used interchangeably [1]. Equating adjusts for differences in difficulty among forms that are built to be similar in difficulty and content. There are several methods of equating two or more tests. Generally, five steps are suggested: First, choose a data collection design. There are two classes of equating designs. First class contains single group or randomly equated group design. Second class contains designs for which the assumption of randomly equivalent groups may not hold. There are three nonequivalent group designs, i.e. common item (anchor item) nonequivalent groups design, pre-equating nonequivalent groups design and post-equating nonequivalent groups design. Second procedure is to get the parametric values such as difficulty index, discrimination index based on the classical test theory or item response theory. Third, common item is selected from two tests with same contents and same format. Fourth, equivalent constant is calculated by scale transformation. The item and ability parameters of two tests are as follows.

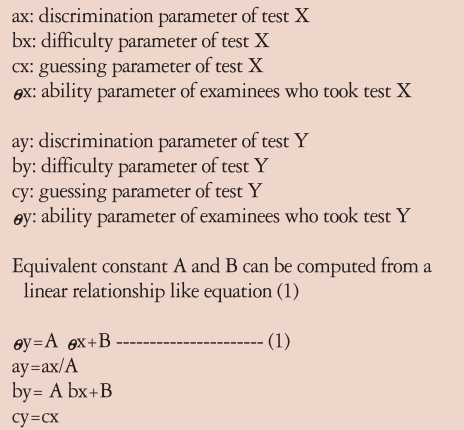



To compute the equivalent constant based on the item response theory; there are regression method, mean and sigma method, robust mean and sigma method, and item characteristic method [2]. Fifth, score equating is possible with common scale ability parameter of two tests. There are true score equating and observed score equating methods for this purpose. If only ability parameters are compared, this last step is not necessary [1].

Out of above procedure, we tried to apply test equating to Medical Licensing Examination for years 2003 and 2004 based on the item response theory that less affected by the ability of examinees. Specifically, we pursued-First, to compare the item parameters post-equating, second to compare the ability parameters post-equating, third, to compare the true score after score equating procedure. Besides answering the origin of the difference in passing rates, this is meaningful not only to suggest the method of comparing yearly results of the Examinations, but also to provide the basis for solving the problem of fluctuation in passing rate every year.

## MATERIALS AND METHODS

The results of the Medical Licensing Examination of one OECD country were reviewed. We let the Examination in 2003 be an old form and 2004 a new form. The number of items in the 2003 Examination was 500 and consisted of General Part (110, 22.0%), Specific organ part (370, 74.0%) and Medical Law (20, 4.0%). The number of items of 2004 Examination was 550 and consisted of General Part (126, 22.9%), Specific organ part (400, 72.7%) and Medical Law (24, 4.4%). The item analysis was completed based on the response of 3,647 examinees in 2003 and 3,879 examinees in 2004. We tried to equate the 2004 Examination to the 2003 Examination.

### Equating procedures

#### 1) Choosing data collection design

Examinees and item contents of two Examinations are not identical. Although there were no same items on the two Examinations, these can be said to be alternate forms since the subjects are the same and the item difficulty index points to an equal level. The common items should be assumed for the two Examinations to equate them. Therefore, common-item nonequivalent design was applied.

#### 2) Estimation of item and ability parameters based on item response theory

The Item Response Theory Command Language (ICL) was used for the estimation of item parameters and ability parameters based on item response theory [3].

#### 3) Selection of common items of two tests

Common items were selected when the contents, knowledge level (recall, interpretation and problem-solving) are identical. Out of them, the items of which difference of difficulty parameters of two years is less than 1, were finally selected. The number of common items was 30.

#### 4) Computing equating constant by scale transformation

We computed the equating constant by scale transformation using a computer program (ST) for IRT Scale Transformation [4]. Two categories of techniques for computing the scale transformation functions are computed in ST: 1) techniques based on the mean and standard deviation of the item parameters; and 2) techniques based on minimizing a loss function involving item characteristic curves.

#### 5) True score equating

We used a computer program for IRT Equating (PIE) to calculate the true score equating that is easier to compute and that does not depend on ability parameters [5]. There is also method of observed score equating. Since there was a report that two methods produce very similar results in a study using the common-item nonequivalent groups design in the SAT, we just tried true score equating [1].

## RESULTS

### Change of item parameters after scale transformation

**Fig. 1:**
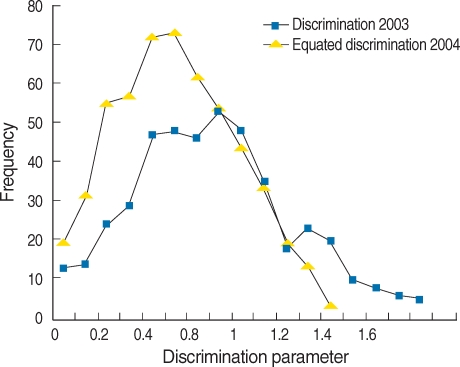
Frequency distribution of discrimination parameter of Medical Licensing Examination analyzed based on item response theory in 2003 and 2004 after scale transformation.

**Fig. 2:**
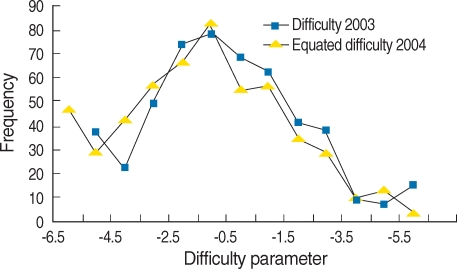
Frequency distribution of difficulty parameter of Medical Licensing Examination analyzed based on item response theory in 2003 and 2004 after scale transformation.

Estimated Values of item parameters of year 2003 Examinations were as follows: Mean discrimination parameter was 0.487 (Standard deviation=SD 0.242), skewness was 0.587 (SD 0.109), and kurtosis was 0.567 (SD 0.218); Mean difficulty parameter was -0.957 (SD 2.682), skewness 0.296 (SD 0.109), kurtosis 0.021(SD 0.218). Values of item parameters of year 2004 before scale transformation were as follows: Mean discrimination parameter was 0.503 (SD 0.269), skewness was 0.767 (SD 0.104), and kurtosis was 1.649 (SD 0.208); Mean difficulty parameter was -1.719 (SD 2.486), skewness 0.623 (SD 0.104), kurtosis 0.772 (SD 0.208). Values of item parameters after scale transformation were as follows: Mean discrimination parameter was 0.363 (SD 0.193), skewness was 0.748 (SD 0.104), and kurtosis was 1.599 (SD 0.208); Mean difficulty parameter was -1.456 (SD 3.399), skewness 0.605 (SD 0.104), kurtosis 0.817 (SD 0.208). The frequency of item characteristics after scale transformation was shown in Figs 1 and 2. The mean of discrimination and difficulty parameters of year 2003 are higher than those 2004. The mean of discrimination parameter of year 2004 after scale transformation decreased from 0.503 to 0.363, meanwhile the standard deviation decreased from 0.269 to 0.193. The mean of difficulty parameter of year 2004 after scale transformation increased from -0.1719 to -1.456 and the standard deviation increased. The Examination of year 2003 can be said to be more difficult to solve and more discriminating than that of year 2004.

### Change of ability parameter after scale transformation

**Fig. 3:**
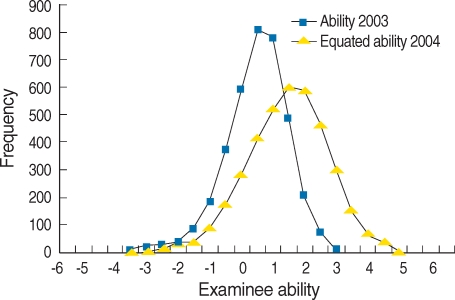
Frequency distribution of ability parameter of examinees who took Medical Licensing Examination analyzed based on item response theory in 2003 and 2004 after scale transformation.

Mean ability parameter in 2003 was 0.00617 (SD 0.96605, skewness was -0.541 (SD 0.041), and kurtosis was 1.099 (SD 0.081). Mean ability parameter in 2004 before scale transformation was -0.00025 (SD 0.95500), skewness -0.411 (SD 0.039), kurtosis 0.820 (SD 0.079). Mean ability parameter in 2004 after scale transformation was 0.94636 (SD 1.32960), skewness -0.411 (SD0.039), kurtosis 0.820 (SD 0.079). The frequency of ability parameters was illustrated in Fig. 3. It shows that the ability of examinees of year 2004 was higher than that of year 2003.

### True score equating

**Fig. 4:**
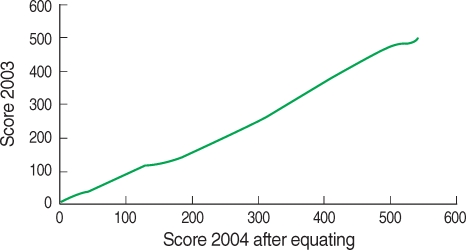
Linear transformation of true score of year 2004 after equating to the score of year 2003 of Medical Licensing Examination.

**Fig. 5:**
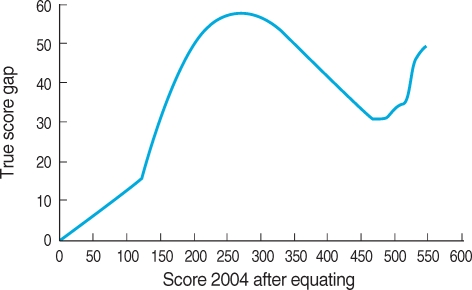
Gap of true score between true score of year 2004 after equating and the true score of year 2003 at the same ability level of Medical Licensing Examination.

The result of true score equating between score of year 2003 and that of year 2004 can be expressed as linear graph (Fig. 4). The scores of year 2003 and 2004 can be matched one to one in the range of ability parameters. This matching is possible from score 0 to maximum score. For example, score 119 in 2003 matched to score 250 in 2004. Fig. 5 shows the discrepancy of the equated true score between two Examinations. It is not linear form. The discrepancy increased linearly at first. From score 150 in 2004, the gap begin to increase. The gap is high in the range of score 200 to 350. It decreases in the range of score 450 to 500. It again soars until maximum score. After equating, the examinees of year 2004 with score 250 can be converted to less score of year 2003. If the score of examinees of year 2004 can be presented with score of examinees of year 2003, the examinees with higher and lesser ability can get advantageous scores than the examinees with middle ability.

## DISCUSSION

Comparing the results of two years' Examinations after scale transformation, year 2003 was more difficult and more discriminating than year 2004. The ability of examinees of year 2004 was higher than that of year 2003. Therefore, the reason for the difference in passing rates over two consecutive years was due to the fact that not only were the items in Examination of year 2004 easier but also the ability of examinees of year 2004 was higher. In addition to the above, the equity of the passing score can be compared. The present passing score is 60% of total score. It was 294 in 2003 and 322.8 in 2004 since the score of Medical Law subject was counted as half score. Seeing the results of equated scores, score 323 in 2004 matches to score 271 in 2003 and score 294 in 2003 matches to score 343-344 in 2004. Therefore, examinees with score between 270 to 294 in 2003 and examinees with score between 322 and 343 in 2004 were affected by the year of Examination. The test equating through common items makes it available to compare the test scores each year, but it is not possible to be compared directly. It also provides the data on the validity of the passing criterion that is inevitably affected by the item difficulty and ability of examinees.

We searched the PubMed (http://pubmed.org), Web of Science(http://isi01.isiknowledge.com/) and Google (http://google.com) to ascertain if there are any comparable equating data of high stake examinations to this paper. No data on the Medical Licensing Examination was searchable. Only Medical College Admissions Test (MCAT) was equated using several methods that mentioned that the item response theory is useful for MCAT [6]. The lack of data on this topic may be due to difficulty of the selection of common items since not every Institute wants to use exposed items.

There are some limitations of this work. First, we cannot use completely-identical common items so that the professionals determined the common items based on the content and knowledge level. Second, the number of common items is short. Thirty is the minimum number of common items when the number of total items is over 150. Since the item numbers are 500 and 550; the number of common items, 30 is the minimum for test equating. Therefore, there may be a possibility of bias originated from the characteristics of common items. In this situation, it is reasonable to say virtual common items instead of common items. There is no available previous work on the virtual common items like ours. In real situation, where completely-identical common items are not available, the introduction of the virtual common items and its results of analysis is another task to be solved.

In the criterion-referenced test, the stable difficulty level of the test is essential. However, if the fluctuation of the passing rate originated from the difference of the abilities of examinees, it is not plausible to say that the difficulty level was not properly arranged. This equating procedure might be invaluable to compare the results of Examinations although they are the alternate forms. If the score can be reported after scale transformation and the equivalent passing score is set, the validity of the test can be obtained more reasonably. The results obtained in this study can be a basis for the computerized adaptive test to input the item parameters of each years Examinations. Test equating can be applied to the elective tests or the objective structured clinical examinations. Work toward the more stable establishment of test equating of Medical Licensing Examination are to continue the yearly comparison of item parameters and ability parameters, to compare the other methods of equating such as equi-percentile methods or observed score equating.
